# Clinical and inflammatory characteristics of patients with asthma in the Spanish MEGA project cohort

**DOI:** 10.1002/clt2.12001

**Published:** 2021-03-27

**Authors:** Manuel J. Rial, Maria J. Álvarez‐Puebla, Ebymar Arismendi, María L. Caballero, José A. Cañas, María J. Cruz, Francisco J. González‐Barcala, Juan A. Luna, Carlos Martínez‐Rivera, Joaquim Mullol, Xavier Muñoz, José M. Olaguibel, César Picado, Vicente Plaza, Santiago Quirce, Christian Romero‐Mesones, Francisco‐Javier Salgado, Beatriz Sastre, Lorena Soto‐Retes, Antonio Valero, Marcela Valverde, Joaquín Sastre, Victora del Pozo

**Affiliations:** ^1^ Servicio de Alergología Departamento de Inmunología Instituto de Investigación Sanitaria (IIS) Fundación Jiménez Díaz Hospital Universitario Fundación Jiménez Díaz Madrid Spain; ^2^ Servicio de Alergología Complejo Hospitalario de Navarra Pamplona Spain; ^3^ CIBER de Enfermedades Respiratorias (CIBERES) Madrid Spain; ^4^ Servicio de Neumología y Alergia Hospital Clínic IDIBAPS Universitat de Barcelona Barcelona Spain; ^5^ Servicio de Alergia Hospital Universitario La Paz IdiPAZ Madrid Spain; ^6^ Departamento de Biología Celular Fisiología e Inmunología Universitat Autónoma de Barcelona Barcelona Spain; ^7^ Servicio de Neumología Hospital Vall d’Hebron Barcelona Spain; ^8^ Servicio de Neumología Complejo Hospitalario Universitario de Santiago Santiago de Compostela Spain; ^9^ Servicio de Neumología Hospital Germans Trias i Pujol Barcelona Spain; ^10^ ENT Department Hospital Clínic IDIBAPS Universitat de Barcelona Barcelona Spain; ^11^ Departamento de Medicina Respiratoria Hospital de la Santa Creu i Sant Pau Instituto de Investigación Biomédica Sant Pau (IIB Sant Pau) Universidad Autónoma de Barcelona. Departamento de Medicina Barcelona Spain; ^12^ Department of Biochemistry and Molecular Biology Faculty of Biology‐Biological Research Centre (CIBUS) Universidade de Santiago de Compostela Santiago de Compostela Spain

**Keywords:** asthma, biomarkers, eosinophilic asthma, inflammation, neutrophilic asthma, phenotype, Spanish: asma; biomarcadores; asma eosinofílica;inflamación; asma neutrofílica; fenotipos.

## Abstract

**Introduction:**

The MEGA (MEchanism underlying the Genesis and evolution of Asthma) project is a multicenter cohort study carried out in eight Spanish hospitals, gathering clinical, physiological, and molecular data from patients with asthma and multimorbidities in order to gain insight into the different physiopathological mechanisms involved in this disorder.

**Material and Methods:**

We report the baseline clinical and physiological characteristics and biomarker measures of adult participants in the project with the aim of better understanding the natural history and underlying mechanisms of asthma as well as the associated multimorbidities across different levels of severity. We carried out a detailed clinical examination, pulmonary function testing, measurement of fractional exhaled nitric oxide (FeNO), blood counts, induced sputum, skin prick tests, chest computed tomography scan, asthma questionnaires, and multimorbidity assessment in 512 asthmatic patients.

**Results:**

When compared to patients with milder disease, severe asthmatic patients showed greater presence of symptoms, more exacerbations, lower asthma control, increased airflow obstruction, and higher frequency of chronic rhinosinusitis with nasal polyps, severe rhinitis, anxiety and depression, gastroesophageal reflux, and bronchiectasis.

**Conclusion:**

The MEGA project succeeded in recruiting a high number of asthma patients, especially those with severe disease, who showed lower control and higher frequency of multimorbidities.

## 1 INTRODUCTION

According to GEMA 5.0,^1^ asthma is a chronic inflammatory disease of the airways in which the pathogenesis involves various cell types and inflammatory mediators. In Spain, the prevalence of asthma ranges from 1.5% to 16.7% in the adult population and is approximately 10% among children.^1^ The current treatment approach consists of stratifying patients by phenotype (clinical, inflammatory, and molecular)^2^ but also by endotype (allergic asthma, nonsteroidal anti‐inflammatory drug [NSAID]‐exacerbated respiratory disease, eosinophilic asthma, or late‐onset asthma), a strategy known as “phenoendotyping.”^3^

Current medications used to treat asthma reduce inflammation of the airways and relieve bronchospasm, but symptoms reappear with cessation of treatment. Recent studies show that over 50% of patients with asthma are not controlled,^1^, ^4^ indicating a need for alternative therapies. Although novel biological treatments directed against type 2 cytokines hold promise,^5^, ^6^ selection of patients most likely to respond to these treatments continues to be hindered by an inadequate understanding of the heterogeneous underlying pathophysiological and molecular mechanisms involved.^7^, ^8^

The present and future research projects should attempt to identify asthma phenotypes using data collected from multiple cohort studies with sufficiently large sample sizes, integrating data from several domains of the disease repeatedly measured over time. The MEchanism underlying the Genesis and evolution of Asthma (MEGA) project^9^ is an ongoing multicenter study in Spain carried out within the framework of the CIBER of Respiratory Diseases. This consortium will perform a number of imaging studies, determinations of lung function, inflammation, and bronchial hyperresponsiveness, and conduct measurements of associated multimorbidities to establish the characteristics that shape this asthma population. In addition, the project will study the stability of different parameters at long term to determine changes in patient condition, exacerbations, control, biomarkers, as well as treatments that can influence the progression of the disease.

The aim of the present study is to describe the baseline characteristics of the adult asthmatic participants that make up the project cohort in terms of their clinical features, frequency of multimorbidities, functional features, and inflammatory biomarkers, so as to better understand the natural history of asthma in patients with different levels of disease severity.

## 2 METHODS

### 2.1 Design

We conducted a multicenter prospective cohort study of 512 adult patients with asthma. Consecutive (unselected) sampling was used to recruit patients from eight university hospitals in Spain.

### 2.2 Data collection

Standard data collection methods were used in all participating research centers. A electronic database and case report form (CRF) were designed to collect study data.^9^

### 2.3 Patient selection and sampling

Asthma severity has been made according to the classification of the Global Initiative for Asthma (GINA).^10^ Asthma diagnosis (based on GINA guidelines) preceded the inclusion of patients by at least 1 year. A standardized clinical history will be completed for each patient and validated Spanish versions of the following questionnaires will be administered: Asthma Control Test (ACT),^11^ Test of Adherence to Inhalers (TAI),^12^ Asthma Quality of Life Questionnaire (Mini‐AQLQ),^13^ the Sino‐Nasal Outcome Test 22 (SNOT‐22),^14^ and the Hospital Anxiety and Depression Scale (HADS).^15^, ^16^ All study subjects will undergo a detailed clinical examination, including body mass index (BMI) and respiratory function tests (baseline spirometry, bronchodilator test, lung volume measurement by plethysmography, fraction of exhaled nitric oxide [FeNO] and CO transfer test [DLCO] using the single‐breath method), following the recommendations of the European Respiratory Society.^17^, ^18^

Methacholine challenge (PC_20_) and induced sputum are performed at baseline and subsequently every 24 months. Chest computed tomography (CT) scan and skin prick tests (SPT) with common aeroallergens were performed at the beginning of the study.

The panel of aeroallergens comprised the following: *Dermatophagoides pteronyssinus*, *Dermatophagoides farinae*, *Lepidoglyphus destructor*, *Alternaria alternata*, *Aspergillus fumigatus*, *Cladosporium herbarum*, *Penicillium notatum*, *Cupressus arizonica*, *Platanus acerifolia*, *Olea europaea*, *Phleum pratense*, *Artemisia vulgaris*, *Parietaria judaica*, *Salsola kali*, *Blatella orientalis*, and epithelia (cat and dog). SPTs will be considered positive at wheal diameters of at least 3 mm compared to the negative control (saline); histamine (10 mg/ml) will be used as a positive control.

Atopy is defined as the presence of at least one positive SPT or aeroallergen‐specific immunoglobulin E (IgE) in serum.

The DNA, serum, exhaled breath condensate, and sputum supernatants have been stored at −80°C in each of the recruiting centers for further analysis. The detailed protocol has been published elsewhere.^9^

### 2.4 Statistical analyses

Comparisons between groups were performed using the unpaired, two‐tailed Student’s *t*‐test for Gaussian samples and Mann–Whitney *U*‐test for non‐Gaussian samples. Normality was analyzed using the Kolmogorov–Smirnov test. Analysis of variance with Bonferroni post hoc test was performed for comparisons between more than two groups of Gaussian samples, and Kruskal–Wallis with Dunn post hoc test was applied for non‐Gaussian distributions. A *p* value less than 0.05 was considered significant. In addition, Spearman's correlation was used to measure the association between clinical parameters.

Statistical calculations and graphs were performed with GraphPad Prism 6 (GraphPad Software Inc.).

In order to determine the contribution of a range of factors (sex, age, BMI, age at onset of asthma, duration of asthma, presence of atopy, and polyposis) to airflow obstruction (FEV_1_/FVC <70% post‐BD), univariate and multivariate regression analyses were performed (Table S2). Regression analysis was performed using SPSS statistical software (IBM Corporate).

## 3 RESULTS

### 3.1 Demographic characteristics

The demographic, functional, clinical, and inflammatory characteristics of the MEGA cohort are summarized in Table 1. Not all data are available for all patients, due to the absence of some data in the CRF, including several comorbidities such as sleep apnea, diabetes, hypertension, or bronchopulmonary mycosis.

**TABLE 1:** Demographic and clinical characteristics of patients included in the MEGA cohort

		*N*	Mean ± *SD*	Intermittent	Mild	Moderate	Severe
Demographic	Age	522	47.3 ± 13	44.08 ± 13.71	42.90 ± 11.3	46.97 ± 12.1	50.50 ± 13
	Female (%)	511	66.15				
	Ethnicity	499					
	Asian (%)		0.002				0.5
	Black (%)		0.002				0.5
	Caucasian (%)		91.99	100	82.2	92.4	94.5
	Hispanic (%)		6.8		16.6	6.4	3.9
	Others (%)		0.78		1.2	1.2	0.5
	Type of delivery at birth	450					
	Dystocic (%)		7.3	13	3.5	7.6	8.2
	Eutocic (%)		92.7	87	96.5	92.4	91.8
	Urban residence during childhood (%)	461	65.6	50	58	69	65
	Pets at home (%)	463	51	58.3	60.23	48.2	50.26
	Age at onset	470					
	<12 years (%)		41.4	30.4	28.7	23.8	28
	12–40 years		20.1	30.4	52.8	46.9	50.1
	>40 years		38.5	39.2	13.5	27.3	21.2
	BMI	496	27.01 ± 5	26.5 ± 5	26.15 ± 4.4	27.35 ± 5.7	27.35 ± 5.4
	Smoking status	486					
	Never		53.2	76.9	71.9	48.2	44.8
	Passive		7.5		3.4	7.6	10.4
	Ex‐smoker		30.8	19.2	19.1	31.8	37.8
	Smoker		8.5	3.8	5.6	12.4	7
	(Mean ± *SD* pack‐years)		35.68 ± 92.5	12	9.75 ± 6.2	57.5 ± 128.1	15.9 ± 15.2
	Alcohol consumer (%)	482	23.3	12	23.9	31.5	17.4
	Athletic activity (%)	481	44.3	24	47.7	41.1	47.5
Inflammatory characteristics	Atopy	481	76.9	76.9	89.9	74.6	73.1
	Total IgE	458	419.1 ± 824	214.4 ± 232.4	30.4 ± 439	427.6 ± 924	492 ± 914
	Eosinophils (cells/mm^3^)	489	336 ± 327	252 ± 174.3	272.5 ± 183	329.4 ± 220	390 ± 444
	Eosinophils in sputum (%)	212	10.49 ± 19	4.72 ± 3.17	6.49 ± 10.7	8.8 ± 16.7	14.11 ± 22.9
	FENO	340	41.82 ± 37	56.63 ± 48.17	42.48 ± 37	36.84 ± 29.6	46 ± 40
Functional parameters	PC_20_ methacholine	198	4.63 ± 10	8.025 ± 12.14	3.073 ± 6	3.72 ± 12.9	4.66 ± 6.3
	RV%	222	123 ± 44	123.2 ± 47.54	132.8 ± 42.5	113.2 ± 42.6	129.4 ± 45.18
	TLC%	227	108 ± 56	95.07 ± 33.66	97.8 ± 28.45	118.2 ± 84.72	104.6 ± 20.32
	FEV1 Pre‐BD (%)	493	85.71 ± 21	102.5 ± 15.8	97.09 ± 15.65	88.18 ± 17.3	75.5 ± 21.8
	FEV1 Post‐BD (%)	365	86.06 ± 3	108.9 ± 16.7	100 ± 26.82	81.92 ± 37.88	81.1 ± 29.3
	FVC Pre‐BD (%)	493	100.1 ± 46	108.9 ± 13.5	107 ± 16.53	101.7 ± 18.48	93.9 ± 68.4
	FVC Post‐BD (%)	364	95.41 ± 59	112.3 ± 12.9	105 ± 28.28	89.5 ± 42.37	94.5 ± 80.3
	FEV1/FVC Pre‐BD	474	71.81 ± 37	112.9 ± 168.6	86.75 ± 97.8	70.16 ± 14.13	67.73 ± 11.70
	FEV1/FVC Post‐BD	333	76.68 ± 65	128.2 ± 198.6	95.96 ± 116.7	72.70 ± 13.3	71.33 ± 12.44
	DLCO	*201*	95.9 ± 22	91.44 ± 14.83	97.8 ± 19.33	96.5 ± 22.35	95.22 ± 23.11
Questionaries	ACT	471	20.27 ± 5	23.70 ± 1.845	21.89 ± 3.74	21.70 ± 3.68	17.97 ± 5.4
	AQL	*444*	*5.48 ± 1.38*	*6 ± 0.84*	5.83 ± 1.33	5.87 ± 1.17	4.92 ± 1.45
	SNOT‐22	416	29.5 ± 19.97	25.22 ± 1.57	24.4 ± 16.84	26 ± 18.9	35.21 ± 21

Abbreviations: ACT, Asthma Control Test; IgE, immunoglobulin E; MEGA, MEchanism underlying the Genesis and evolution of Asthma; RV, residual volume; SNOT‐22, Sino‐Nasal Outcome Test 22; TLC, total lung capacity.

A total of 512 patients were included in the study (66% women). Most patients were Caucasian (92%). Obesity, defined as a BMI > 30, was found in 25% of patients (*n* = 124); of these, 84% (*n* = 104) had severe persistent asthma and 16% (*n* = 20) intermittent or mild persistent asthma (*p* = 0.0269). Currently, 88% of the patients live in an urban setting, but when these same patients were children this percentage was lower (65.6%), and significantly higher in the most severe asthma patients (*p* = 0.054). Fifty percent of patients had a first‐degree relative with a history of asthma. As for smoking, 53.4% of patients were nonsmokers, 31% ex‐smokers, 8.2% smokers, and 7% passive smokers. Asthma severity is not related with smoking habit. Occupational asthma was found in 7.7% of patients, and 13.4% had work‐related asthma.

**FIGURE 1:**
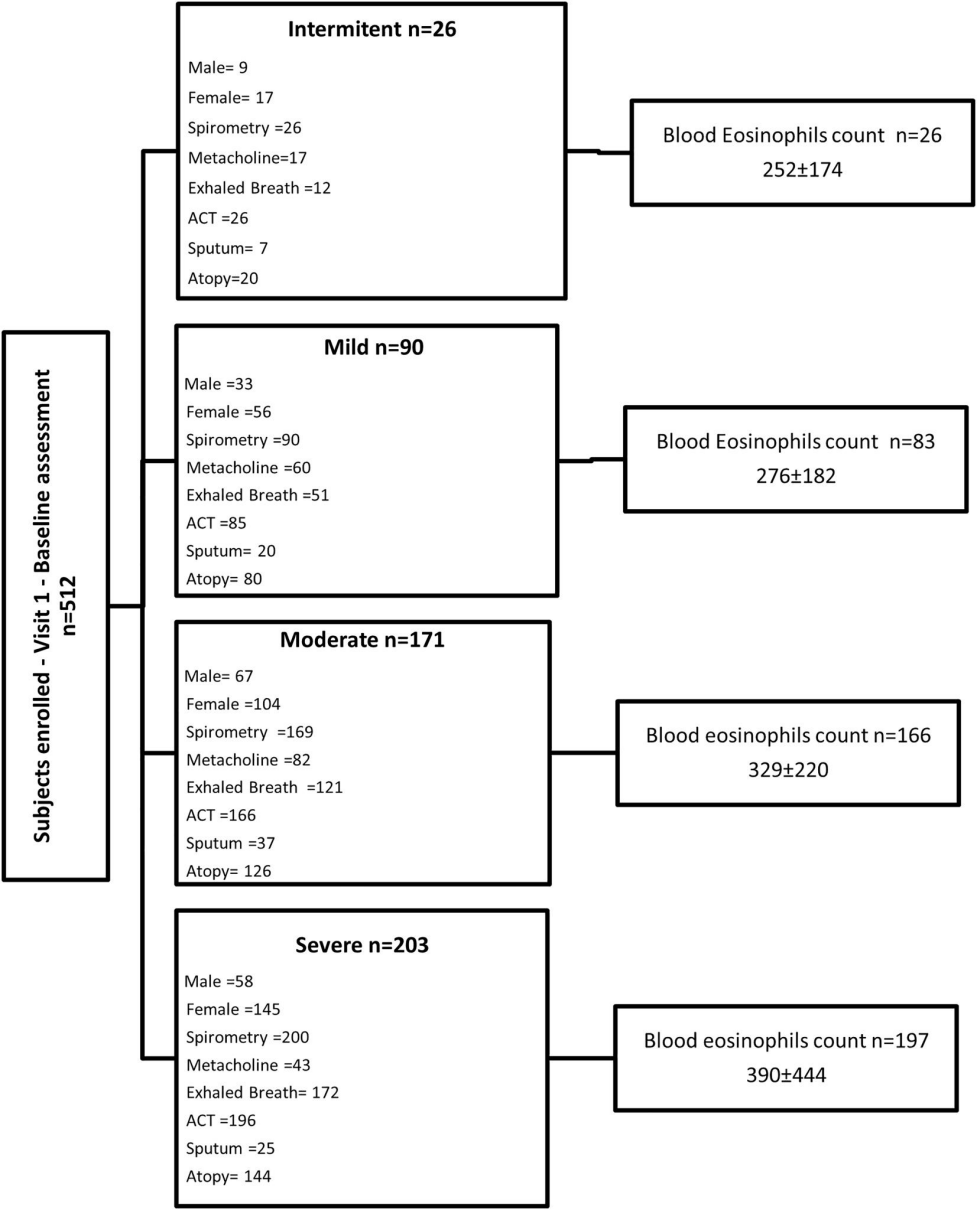
Flow chart showing visit schedule. The items appearing in the boxes are defined as follows: Sex; number of patients who completed prebronchodilator spirometry, a specific inhalation challenge with methacholine, exhaled breath condensate, the Asthma Control Test (ACT), induced sputum. Atopy is defined by at least one positive skin prick test (SPT) or detectable levels of specific IgE for any allergen tested. IgE, immunoglobulin E

Regarding the severity of asthma, 5% of patients had intermittent asthma (*n* = 26), 17% mild asthma (*n* = 90), 33.4% moderate asthma (*n* = 171), and 39.6% severe asthma (*n* = 203) (Figure 1). A total of 22 patients (4.3%) could not be correctly classified due to lack of information in the electronic registry. Bronchiectasis was present in 7% of patients (*n* = 36), with 67% of them (*n* = 24) experiencing persistent severe asthma. The age of severe patients is significantly higher than those with less severity.

**FIGURE 2:**
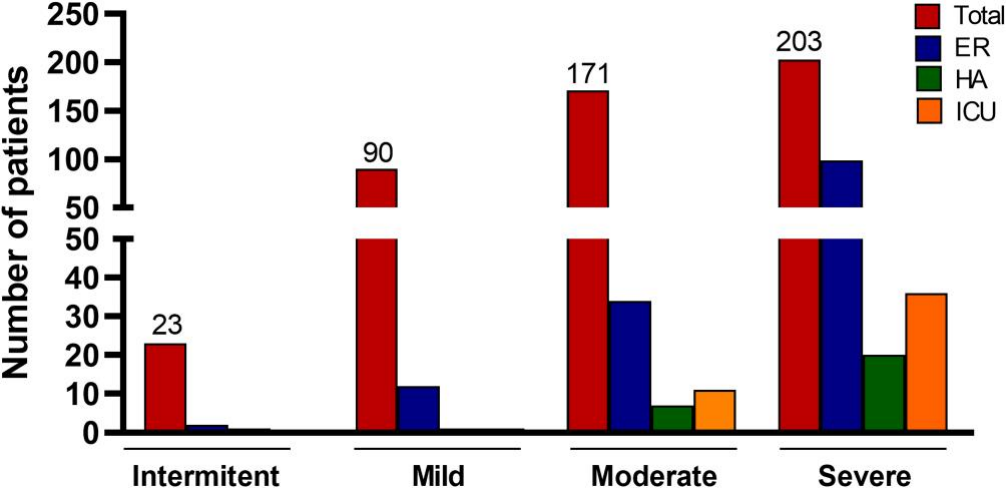
Patients with severe exacerbations according to asthma severity and hospital admissions. ER, emergency room admission; HA, hospital admissions; ICU, intensive care unit admission

Over the previous year, 15.5% of patients had developed more than three exacerbations, and only 1.4% did not present any exacerbations in this period. Of all patients who experienced exacerbations, 18.6% required hospital admission during the last year, and 9.8% required at least one admission in the intensive care unit (Figure 2).

Of the 74 patients diagnosed with NSAID hypersensitivity, 54% (*n* = 40) had severe asthma (*p* < 0.001). Of the 24 patients diagnosed with food allergy, 37.5% (*n* = 9) have severe asthma and 42% (*n* = 10) moderate persistent asthma.

### 3.2 Inflammatory characteristics

**FIGURE 3:**
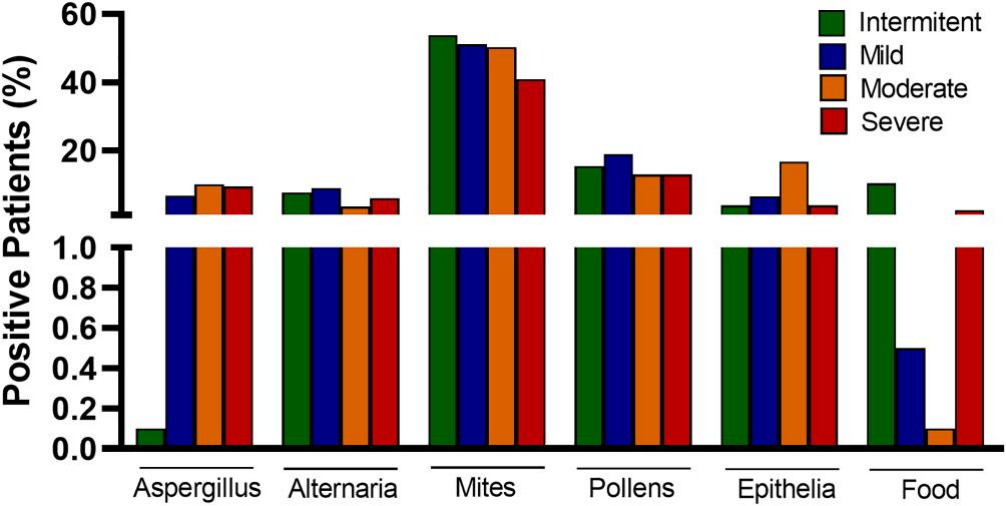
Sensitization to aeroallergens and asthma severity levels

Among the studied patients, 72.3% (*n* = 370) had at least one positive SPT to the aeroallergen tested. Atopy is significantly lower in moderate and severe asthma compared to mild asthma (*p* = 0.032 and *p* = 0.011, respectively). One hundred eighteen patients (23%) had positive SPT to dog, 22% (*n* = 110) to cat, 45% (*n* = 230) to mites, 15% (*n* = 76) to pollen, 8% (*n* = 42) to Aspergillus, and 5.5% (*n* = 28) to Alternaria (Figure 3).

The level of IgE was 419.1 ± 824 and tended to increase at greater degrees of severity.

Also, 52% of patients (*n* = 266) had allergic rhinitis, 10% (*n* = 52) atopic dermatitis, 18% (*n* = 90) allergic conjunctivitis, 29% (*n* = 148) had chronic rhinosinusitis with nasal polyps (CRSwNP), 14.5% (*n* = 74) showed hypersensitivity to NSAIDs, and 4.7 % (*n* = 24) were diagnosed with food allergy. Of the 148 patients with CRSwNP, only 1 patient had intermittent asthma, 10 mild asthma, 49 moderate asthma, and 88 (59%) presented severe asthma (*p* < 0.001).

**FIGURE 4:**
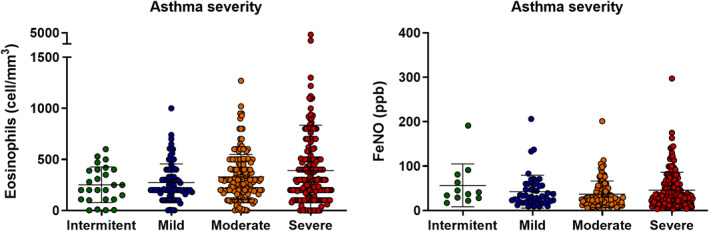
Blood eosinophil count and FENO levels (expressed as mean/median and standard error). No statistically significant differences (*p* < 0.05) were found between blood eosinophil counts and FeNO levels and the severity of asthma

Peripheral blood eosinophilia was measured in all patients at the start of the study (Table 1 and Figures 1 and 4). Eighty percent of patients (*n* = 409) had a peripheral eosinophil count of ≥150 cells/mm^3^, 53.3% (*n* = 273) had a count that was equal to or higher than 300 cells/mm^3^, 38.8% (*n* = 197) had a count of ≥400 cells/mm^3^, and 28.3% (*n* = 145) had levels above 500 cells/mm^3^ (Figure 3). A correlation was found between eosinophil levels above 500 and the presence of atopy (*p* = 0.0375, relative risk = 0.6884, Katz's approximation). There were no significant differences in eosinophil or FeNO levels according to asthma severity (Figure 4), although a tendency toward increased eosinophil counts was seen in severe asthma compared to intermittent disease (1.5‐ and 3‐fold in blood and sputum, respectively).

Induced sputum was available for 212 patients. An eosinophilic inflammatory profile (defined as >3% of eosinophils and <61% of neutrophils) was found in 64 patients (30.2%), a neutrophilic profile (<3% of eosinophils and >61% of neutrophils) in 25 patients (11.8%), a paucigranulocytic pattern (defined as <61% neutrophils and <3% of eosinophils) in 52 patients (24.5%), and we observed a mixed pattern (>3% eosinophils and >61% neutrophils) in 64 patients (30.2%).

**FIGURE 5:**
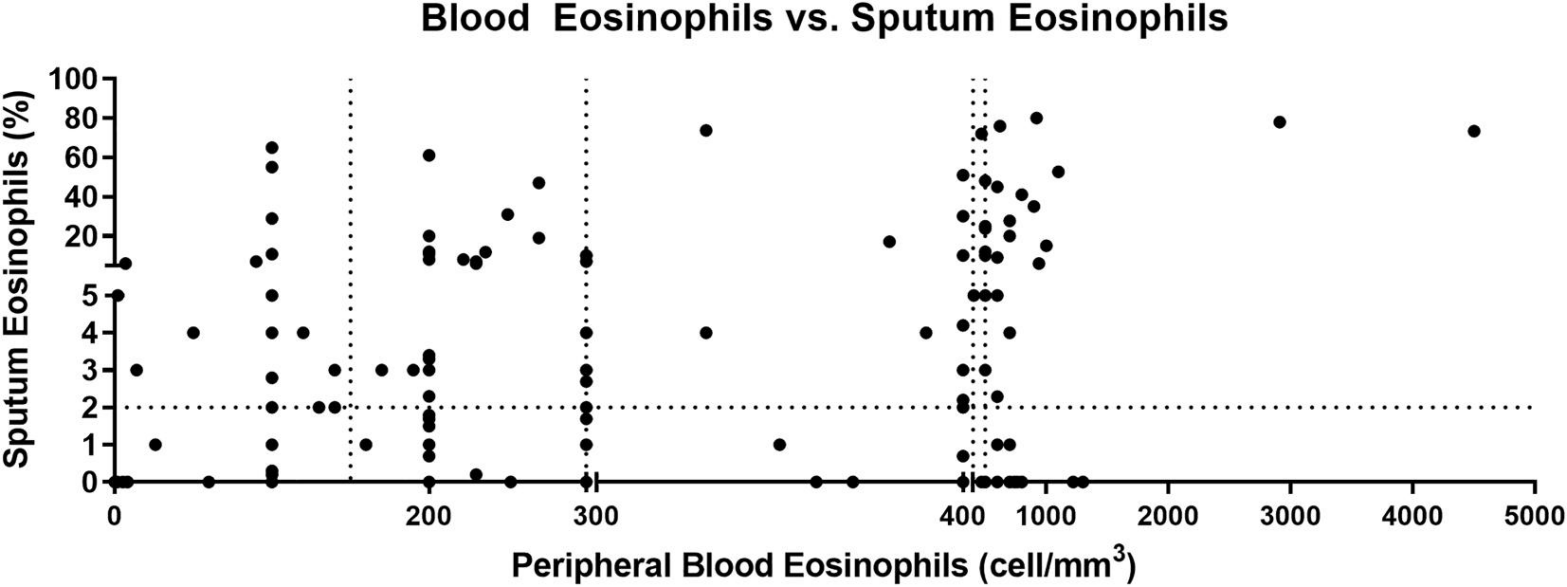
Correlation between eosinophilia in peripheral blood and sputum. The best correlation is established between more than 300 cells/mm^3^ in peripheral blood and more than 2% of eosinophils in sputum (correlation coefficient = 0.5235, *p* = 0.0002)

Correlations have been made with different levels of eosinophilia in peripheral blood (150, 300, 400, and 500 cells/mm^3^) and in sputum (>2% and >3%). The results show that there are more patients with above 300 cells/mm^3^ eosinophilia in sputum, as shown in Figure 5. The best correlation is established between more than 300 cells/mm^3^ in peripheral blood and more than 2% eosinophils in sputum (Spearman *r* = 0.5235, *p* = 0.0002).

### 3.3 Functional parameters

**FIGURE 6:**
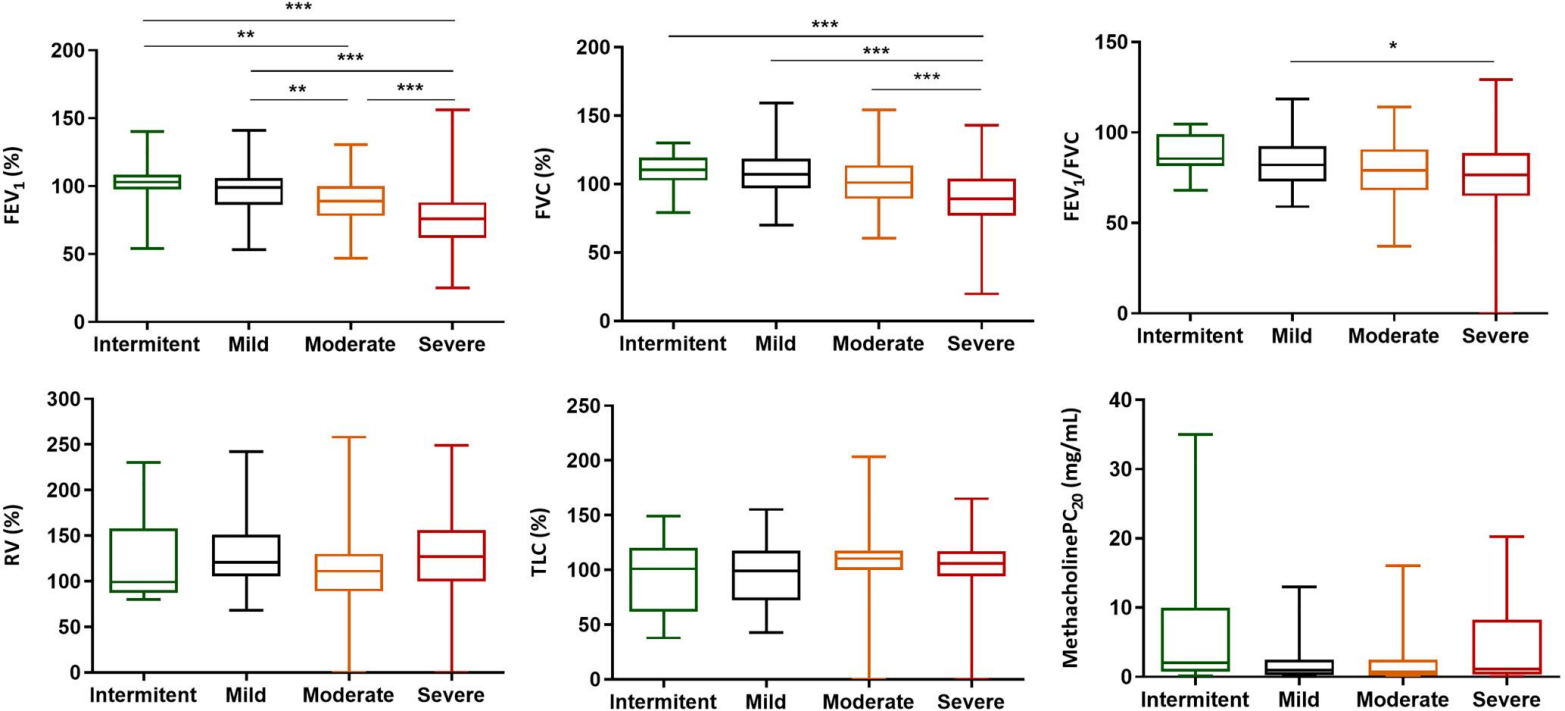
Spirometric, pletismography, and bronchial hyperreactivity data. There are statistically significant differences between FEV1%, FVC%, FEV1/FVC, and asthma severity levels *p < 0.05, **p < 0.01, ***p < 0.001. No statistically significant differences were found in FVC%, RV%, TLC%, and PC_20_ methacholine and asthma severity. RV, residual volume; TLC, total lung capacity

Lung function test results and different asthma severity levels are shown in Figure 6. Statistically significant differences have been found between spirometry values (FEV1%, FVC%, FEV1/FVC) and the different levels of asthma severity. No statistically significant differences were found in plethysmography values (residual volume [RV], total lung capacity [TLC]%), DLCO, or in bronchial hyperresponsiveness with methacholine.

### 3.4 Questionnaires for disease control, quality of life, and anxiety/depression

**FIGURE 7:**
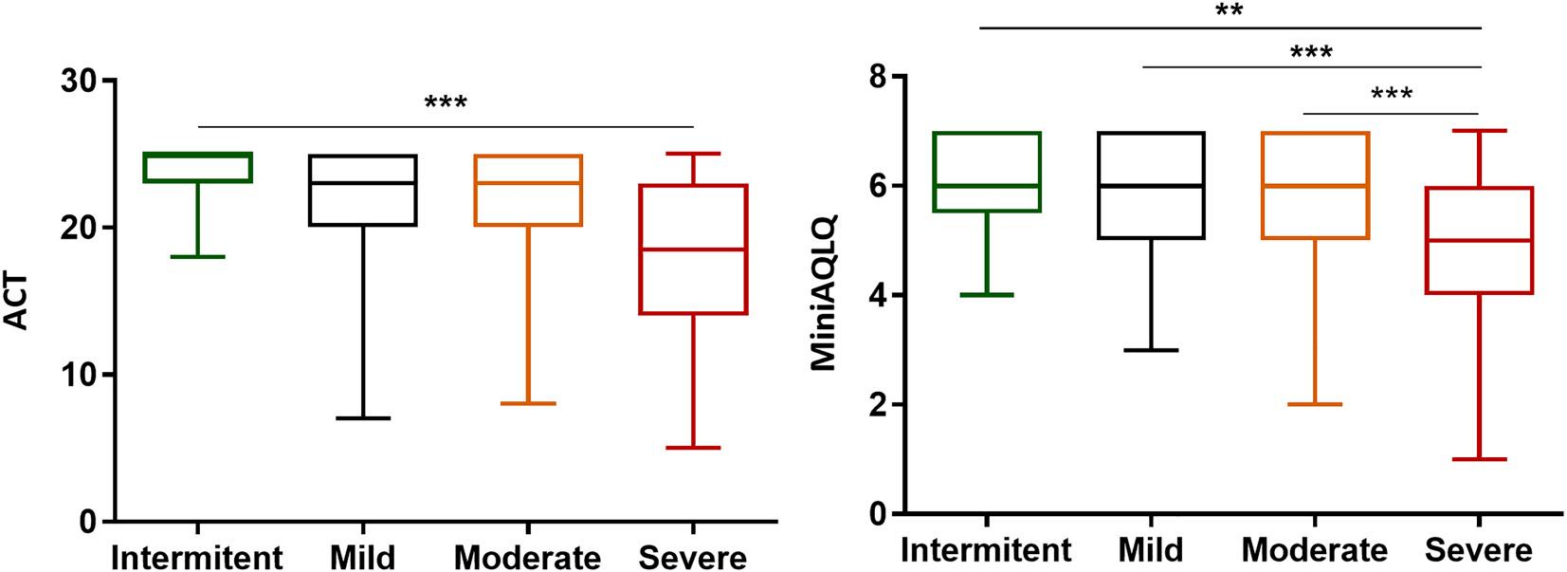
ACT and Mini‐AQLQ scores. Patients with severe asthma (191) had worse control with an average ACT score of 18 points (*p* < 0.001). Statistically significant differences *p < 0.05, **p < 0.01, ***p < 0.001 were found between different levels of asthma severity. ACT, Asthma Control Test; AQLQ, Asthma Quality of Life Questionnaire

ACT scores of <20 were recorded for 34.5% of patients (*n* = 134). The Mini‐AQLQ questionnaire showed a mean score of 5.48 ± 1.38. These results are reflected in Figure 7 and Table 1.

Correlations were made with the Fisher test to determine whether patients with ACT levels <20 had more exacerbations in the previous year, and a statistically significant association was found (*p* < 0.0001).

Anxiety and depression were assessed using the HADS Questionnaire, revealing statistically significant differences (*p* < 0.001) between patients with severe asthma and other severity levels. Patients with severe disease had scores compatible with depression in 17%, and scores for 39% of patients were compatible with anxiety. Patients with moderate persistent asthma had values compatible with depression in 8.9% of patients and with anxiety in 37.8% of patients; among patients with intermittent and mild persistent asthma, 8% had levels compatible with depression and 24% that were compatible with anxiety.

We performed the SNOT‐22 test, comparing the results across different levels of asthma severity. Statistically significant differences were found (*p* < 0.001), with higher SNOT‐22 values associated with more severe asthma.

Adherence to therapy was also measured using the TAI test, revealing somewhat poor or poor adherence in 18% of patients.

### 3.5 Treatments

During the study period, 386 patients were using an inhaled corticosteroid (ICS)/long‐acting beta2‐agonist combination, 24% (*n* = 95) were using a metered‐dose inhaler, and more than half of them used a spacer. Seventy‐six percent (*n* = 291) of the subjects used dry powder devices: 47.07% (*n* = 137) Turbuhaler, 22% (*n* = 64) Accuhaler; 14.4% (*n* = 42) Ellipta, 13.4% (*n* = 39) Nexthaler, and 4% used other devices. In all patients, data concerning the inhalation technique was obtained by direct observation, and 97% of patients rated their treatment as adequate (*n* = 373).

Regarding the treatment with biological drugs, there are 95 patients treated with a biological medication, that is, 73% with omalizumab (*n* = 69), 19% with mepolizumab (*n* = 18), 4% with reslizumab (*n* = 4), and another 4% (*n* = 4) with benralizumab.

## 4 DISCUSSION

This is the largest cohort of Spanish asthmatic patients studied to date. Overall, the clinical characteristics of these patients are consistent with those found in the European cohort U‐Biopred^19^ as well as other large asthma cohorts described worldwide.^8^, ^20^, ^21^, ^22^, ^23^, ^24^ Patients included in this, the MEGA study, showed a clearly significant association between severe asthma on the one hand and increased symptoms and exacerbations, lower disease control, greater airflow obstruction and higher frequency of CRSwNP, severe rhinitis, anxiety and depression, gastroesophageal reflux, and bronchiectasis on the other when compared to patients with milder disease.

A number of findings make this cohort of asthmatic patients one of great interest. Significant associations were found between airflow obstruction and various demographic and clinical features, such as age, asthma duration, and the presence of CRSwNP (Table S2). These data contrast with those of other cohorts, in which a strong association was found with female sex, BMI, and presence of atopy.^24^ In our cohort, these variables seem to be associated with airflow obstruction, but do not have predictive ability, since the adjusted *R* ^2^ is only 3.4%. Random forest modeling was used to complete the regression analysis, though this did not improve predictability. It will be interesting to know whether these associations change over the follow‐up period and whether the predictive capacity of these associations can be assessed.

Air trapping is a characteristic of the severely asthmatic population, and the RV rises with increasing severity.^25^ Furthermore, air trapping has been more frequently associated with neutrophilic phenotypes and poor response to ICS with the presence of persistent bronchial obstruction. In our population, however, there was no statistical difference across patients in terms of RV or TLC according to severity classification.

A novel aspect of this study is the evaluation of rhinosinusitis by means of the SNOT‐22 questionnaire. As expected, we found that higher SNOT scores (more severe rhinosinusitis) were correlated with more severe asthma. It is also important to note that NSAID intolerance was strongly associated with asthma severity in this cohort.

It was not possible to calculate the average dose of ICS that patients receive due to a wide variety of devices and formulations available in the Spanish market. However, all patients were classified by severity according to the medication needed to achieve asthma control. Patients with mild asthma required low doses (200–400 μg/day of budesonide or equivalent), while moderate asthma required moderate doses (401–800 μg/day of budesonide or equivalent), and severe patients required high doses (801–1600 μg/day). We were also unable to measure the average dose of systemic corticosteroids due to the different corticosteroid presentations used as well as the use of depot formulations.

When interpreting these data, it is important to note the good results obtained with regard to the inhalation technique (adequate in 97%) and adherence (adequate in 82%) in comparison to other cohorts.^8^, ^19^, ^20^, ^21^, ^22^ However, these results may be due to the fact that the patients were recruited in specialized centers and may not reflect real life in primary care, where treatment is often inadequate.^26^

We found elevated peripheral blood eosinophil counts in 53.3% of patients, as indicated by mean values above 300 cells/mm^3^, and 28.3% had levels above 500 cells/mm^3^. An assessment of eosinophilic inflammation based on differential cell count in induced sputum samples revealed that 73% of the patients presented more than 2% of eosinophils, indicating levels above other cohorts described in the literature.^19^, ^22^, ^27^, ^28^ We investigated whether an eosinophil count of more than 400 cells/mm^3^ is a risk factor for having worse asthma control (ACT < 20), though no statistically significant differences were detected. Furthermore, no differences were found between patients with eosinophil counts above or below 400 cells/mm^3^ and early‐onset disease debuting in childhood or adolescence. Furthermore, we have found a positive correlation between the levels of eosinophilia in blood and in sputum when the cut‐off point of eosinophils in sputum was set at more than 2% and in peripheral blood higher than 300 cells/mm^3^, a better correlation index was obtained than establishing the sputum cut‐off point at more than 3% (correlation index 0.5235 vs. 0.4819)**.** The latter may be due to the high proportion of patients with CRSwNP (29%) and atopy included in the sample (71%). Blood eosinophil counts above 500 cells/mm^3^ correlated with the presence of atopy. It is important to note that in this cohort, eosinophil levels are not associated with asthma control or age at disease onset.

In asthma, sensitization to fungal, perennial, or seasonal allergens increases the risk of uncontrolled symptoms, exacerbations, and poor disease outcomes.^10^ In severe asthma, 20% to 29% of patients typically show sensitization to ≥1 fungal allergen, with Aspergillus being one of the most common.^29^ These patients have worse lung function, increased risk of oral corticosteroid use and hospitalization, and a higher degree of airflow obstruction than patients not sensitized to fungal allergens.^30^ Nevertheless, our patients presented a similar rate of sensitizations to Aspergillus and Alternaria at different severities. A similar finding was observed with other allergens.

The study has a number of limitations, such as the lack of a control group and the fact that patients were recruited in specialized centers with a higher number of severe asthma patients than mild cases. In addition, the diagnosis of CRSwNP was based on data obtained from the clinical history.

To conclude, among the patients with asthma included in this cohort, eosinophilic asthma was the predominant phenotype, and many were atopic. An increase in disease severity was associated with a higher number of comorbidities, more exacerbations, worse disease control, a greater tendency to experience anxiety and depression, and worse lung function. The characteristics of the patients included are consistent with those previously reported in other cohorts. This cohort is useful for characterizing different asthma phenotypes and in identifying associated biomarkers as well as the stability of these biomarkers over time.

Continued follow‐up of these patients will shed light on the long‐term factors that may influence disease severity and will likely provide insight into the treatments that can influence the progression of the disease, identifying the possible causes of the exacerbations and how such exacerbations affect the clinical course of the disease.

## CONFLICT OF INTERESTS

Dr. Rial reports nonfinancial support from CIBER de Enfermedades Respiratorias (CIBERES), during the conduct of the study; personal fees from GSK, personal fees from Allergy Therapeutics, personal fees from AstraZeneca outside the submitted work. Dr. González Barcala reports personal fees from ALK, personal fees from AstraZeneca, personal fees from Bial, personal fees from Boehringer Ingelheim, personal fees from Chiesi, personal fees from Gebro Pharma, personal fees from GlaxoSmithKline, personal fees from Laboratorios Esteve, Menarini, Mundipharma, Novartis, Rovi, Roxall, Stallergenes‐Greer, Teva, and Grants from Mundipharma outside the submitted work. Dr. Quirce reports personal fees from AstraZeneca, personal fees from Novartis, personal fees from Sanofi, personal fees from Boehringer Ingelheim, personal fees from Teva, personal fees from ALK, personal fees from Mundipharma, personal fees from GSK, personal fees from Chiesi, personal fees from Leti, outside the submitted work. Dr. Romero‐Mesones reports personal fees from Teva, personal fees from GSK, outside the submitted work. Dr. Soto‐Retes reports non‐financial support from CIBER de Enfermedades Respiratorias (CIBERES), during the conduct of the study; personal fees from Stallergennes‐Greer, personal fees from Menarini, personal fees from Novartis, personal fees from GSK, personal fees from Hal Allergy, personal fees from Allergy Therapeutics, personal fees from AstraZeneca, grants from Sociedad Española de Alergologia e Inmunología Clinica SEAIC, grants from Sociedad Española de Neumologia y Cirugia Torácica SEPAR, outside the submitted work. Dr. Martinez Rivera reports grants and personal fees from AstraZeneca, grants and personal fees from Teva, grants and personal fees from GSK, personal fees from Novartis, personal fees from Mundipharma, outsider the submitted work. Dr. Munoz reports personal fees from AstraZeneca, personal fees from Boehringer Ingelheim, grants and personal fees from GlaxoSmithKline, grants from Menarini, personal fees from Novartis, personal fees from Teva, personal fees from Mundifarma, grants and personal fees from Chiesi, personal fees from Faes, outside the submitted work. Dr. Sastre reports grants from Sanofi, during the conduct of the study; grants and personal fees from Sanofi, personal fees from GSK, personal fees from Novartis, personal fees from AstraZeneca, personal fees from Mundipharma, personal fees from Faes Farma, outside the submitted work. Dr. Luna‐Porta reports other from Astra Zeneca, outside the submitted work. Dr. Olaguibel reports grants from Sanofi during the conduct of the study; personal fees from AstraZeneca, personal fees from Mundipharma, outside the submitted work. Dr. Mullol reports personal fees and other from Sanofi‐Genzyme and Regeneron, personal fees and other from Genentech and Novartis, grants and personal fees from Mylan Pharma, grants and personal fees from Uriach Group, personal fees from Mitsubishi Tanabe, personal fees from Menarini, personal fees from UCB Pharma, personal fees from AstraZeneca, personal fees from GSK, personal fees from MSD, outside the submitted work. Dr. Plaza reports grants and personal fees from AstraZeneca, personal fees from Boehringer Ingelheim, personal fees from Merck, grants and personal fees from Chiesi, personal fees from Novartis, grants from Menarini, personal fees from Sanofi, outside the submitted work. Other authors have no conflicts of interests.

## AUTHOR CONTRIBUTIONS

Manuel J. Rial analyzed the patient data and was a major contributor in writing the manuscript. Joaquín Sastre and Victora del Pozo interpreted the patient data and designed the statistical study. All the authors have provided data to be able to carry out the study. All authors read and approved the final manuscript.

## Supporting information

TABLE S1
