# Supplementary material for: Clinical and inflammatory characteristics of patients with asthma in the Spanish MEGA project cohort
Source: Clin Transl Allergy. 2021 Mar 27;11(1):e12001. doi: 10.1002/clt2.12001 (PMC8173588; doi:10.1002/clt2.12001)
Supplement: Supplementary file 1 — TABLE S1 [file CLT2-11-e12001-s001.docx]

**Online repository Table II**: Results of regression analysis. Dependent variable: airflow obstruction (FEV1 / CVF post-BD <70).

a-) Univariate analysis

| Variable | Coef. | (95% CI) | P |
| --- | --- | --- | --- |
| Age | 0.996 | (0.994, 0.998) | <0.001 |
| Sex | 0.988 | (0.935, 1.043) | 0.651 |
| Neonatal history | 1.012 | (0.946, 1.082) | 0.730 |
| History of childhood asthma | 0.987 | (0.937, 1.041) | 0.637 |
| Duration of asthma | 0.998 | (0.996, 1.000) | 0.031 |
| BMI | 0.997 | (0.992, 1.002) | 0.186 |
| SPT and/or CAP | 0.991 | (0.933, 1.054) | 0.778 |
| CRSwNP | 0.930 | (0.879, 0.984) | 0.011 |

b-) Multivariate analysis.

| Variable | Coef. | (95% CI) | P |
| --- | --- | --- | --- |
| Age | 0.996 | (0.994, 0.998) | 0.001 |
| Sex | 0.983 | (0.931, 1.038) | 0.535 |
| Neonatal history | 0.996 | (0.927, 1.071) | 0.914 |
| History of childhood asthma | 0.972 | (0.915, 1.033) | 0.361 |
| Duration of asthma | 0.999 | (0.998, 1.001) | 0.532 |
| BMI | 0.999 | (0.994, 1.004) | 0.714 |
| SPT and/or CAP | 0.959 | (0.900, 1.022) | 0.201 |
| CRSwNP | 0.942 | (0.889, 0.998) | 0.042 |
